# Histone deacetylases in the regulation of cell death and survival mechanisms in resistant BRAF-mutant cancers

**DOI:** 10.20517/cdr.2024.125

**Published:** 2025-01-25

**Authors:** Bernhard Biersack, Bianca Nitzsche, Michael Höpfner

**Affiliations:** ^1^Organic Chemistry Laboratory, University Bayreuth, Bayreuth 95440, Germany.; ^2^Institute of Physiology, Charité-Universitätsmedizin Berlin, Corporate Member of the Freie Universität Berlin, Humboldt-Universität zu Berlin and Berlin Institute of Health, Berlin 10117, Germany.

**Keywords:** Apoptosis, autophagy, BRAF-mutant cancer, drug resistance, histone deacetylase, melanoma, senescence, sirtuin

## Abstract

Small-molecule BRAF inhibitors (e.g., vemurafenib and dabrafenib) and MEK (MAPK/ERK) kinases inhibitors (e.g., trametinib) have distinctly improved the survival of patients suffering from BRAF-mutant cancers such as melanomas. However, the emergence of resistance to BRAF and MEK inhibitor-based melanoma therapy, as well as the reduced sensitivity of other BRAF-mutant cancers such as CRC, poses a considerable clinical problem. For instance, the reactivation of MAPK/ERK signaling hampering cell death induction mechanisms was responsible for BRAF inhibitor resistance, which can be correlated with distinct post-translational and epigenetic processes. Histone deacetylases (HDACs) are prominent epigenetic drug targets and some HDAC inhibitors have already been clinically approved for the therapy of various blood cancers. In addition, several HDACs were identified, which also play a crucial role in the drug resistance of BRAF-mutant cancers. Consequently, inhibition of HDACs was described as a promising approach to overcome resistance. This review summarizes the influence of HDACs (Zn^2+^-dependent HDACs and NAD^+^-dependent sirtuins) on BRAF-mutant cancers and BRAF inhibitor resistance based on upregulated survival mechanisms and the prevention of tumor cell death. Moreover, it outlines reasonable HDAC-based strategies to circumvent BRAF-associated resistance mechanisms based on downregulated cell death mechanisms.

## INTRODUCTION

Mutations of the BRAF (B-Raf, B-rapidly accelerated fibrosarcoma) serine-threonine kinase are associated with poor prognoses in melanoma, colorectal cancer (CRC), non-small cell lung cancer (NSCLC), and other cancers^[1]^. As a crucial component of the Ras (rat sarcoma)-MAPK (mitogen-activated protein kinase)/ERK (extracellular signal-regulated kinase) signaling pathway, which is strongly activated in numerous cancers, BRAF conveys activating signals from receptor tyrosine kinases (RTKs) and activated Ras to downstream MEK (MAPK/ERK) kinases and ERKs via phosphorylation^[2]^. V600E mutation associated with drug resistance, tumor growth and invasiveness is the most relevant BRAF mutation, occurring in 90% of all BRAF-mutant cases^[3]^. Targeting Ras-MAPK signaling is an attractive strategy in cancer therapy; however, drug resistance limits the clinical application of kinase inhibitors such as BRAF and MEK inhibitors^[4,5]^. The resistance to BRAF inhibitor therapy is associated with impaired cell death induction and can be overcome by tackling MAPK reactivation and/or bypassing signaling pathways regulated by various RTKs^[6]^. Further relevant drug resistance mechanisms in BRAF-mutant cancers include phenotype switching (MITF/WNT5 pathways), immunomodulation and changes in the tumor microenvironment, metabolic rewiring (glucose, glutamine, lipid and mitochondrial metabolisms), and epigenetic modifications (histone and DNA modifications, non-coding RNAs)^[7]^. The evasion of apoptosis induction is a hallmark of BRAF inhibitor-resistant BRAF-mutant melanoma, and epigenetic mechanisms play a vital role in the prevention of cell death during BRAF inhibitor therapy^[8]^.

The identification of eminent factors involved in the epigenetic regulation of Ras-MAPK and its bypassing pathways has garnered importance in anticancer drug design and development^[9]^. The temporary modification of histones by acetylation and methylation has consequences on histone-DNA interaction and gene transcription, and plays a crucial role in cancer development and progression^[10]^. While histone acetyltransferases (HATs) add acetyl groups (from acetyl-CoA) to the ε-amino group of lysines of histones and other proteins, histone deacetylases (HDACs) remove these lysine modifications^[11]^. Vital non-histone proteins also undergo lysine acetylation and regulation by HATs and HDACs^[12]^. Thus, the development of HDAC inhibitors has become a valuable strategy to treat cancer and other human diseases. Several small-molecule HDAC inhibitors were approved for the therapy of blood cancers, while their application for solid tumors experienced several drawbacks^[13]^. Meanwhile, the combination of HDAC inhibitors with other anticancer drugs led to promising clinical results, which might pave the way for the application of this class of epigenetic drugs against solid tumors in the future^[14,15]^. In line with this development, there is also growing evidence for the positive effects of HDAC inhibitors on BRAF-mutant cancers^[16]^. The role of HDACs in resistance through impaired cell death and promoted cell survival in BRAF-mutant cancers is outlined in this review.

## ROLE OF BRAF AND HDACS IN CANCERS

### BRAF and its inhibitors

#### An overview of BRAF biology

**Figure 1:**
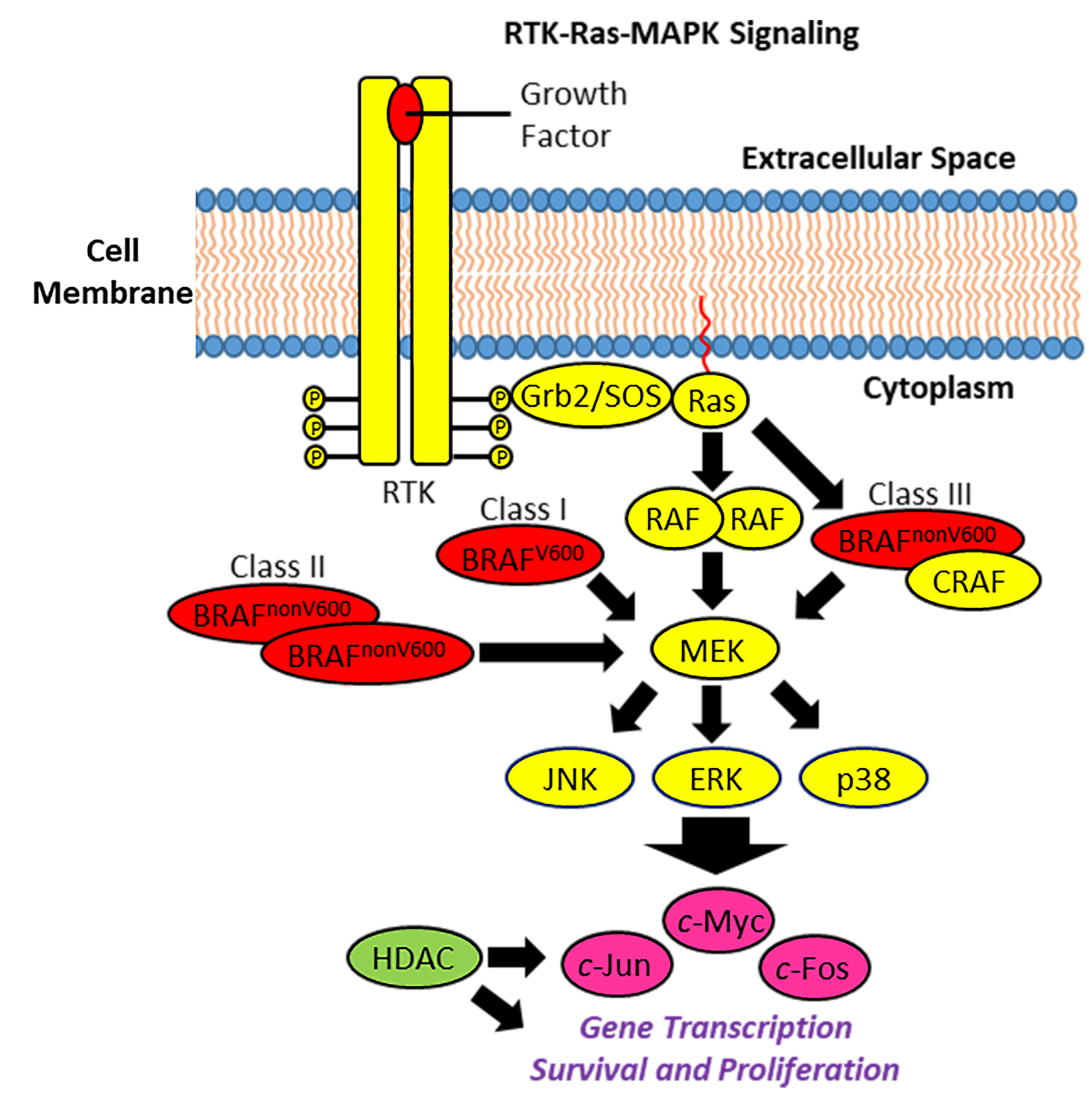
RTK-Ras-MAPK signaling, BRAF mutants and HDAC influence. Binding to the corresponding growth factor activates the RTK, leading to receptor dimerization and autophosphorylation. Docking and adapter proteins (Grb2-SOS complex) recognize and bind the phosphorylated RTK, whereupon farnesylated Ras is activated. Ras activates RAF, which dimerizes and phosphorylates MEKs, followed by phosphorylation of MAPKs (JNK, p38) and/or ERKs. Activated MAPK/ERK upregulated oncogenic transcription factors (e.g., *c*-Jun, *c*-Fos, and *c*-Myc), which promote cell proliferation and survival. HDACs regulate gene transcription. Mutant BRAFs (class I BRAF^V600^ monomers, class II BRAF^nonV600^ homodimers, and class III BRAF^nonV600^-CRAF heterodimers) activate MAPK signaling in uncontrolled/dysregulated ways. ERK: Extracellular signal-regulated kinase; Grb2: growth factor receptor-bound protein 2; HDAC: histone deacetylase; JNK: *c*-Jun kinase; MAPK: mitogen-activated protein kinase; MEK: MAPK/ERK kinase; RAF: rapidly accelerated fibrosarcoma; Ras: rat sarcoma; RTK: receptor tyrosine kinase; SOS: son of sevenless; BRAF: B-Raf, B-rapidly accelerated fibrosarcoma.

Raf kinases (ARAF, BRAF, and CRAF) are serine/threonine kinases and are part of the Ras-MAPK/ERK signaling pathway [Figure 1]^[2]^. Extracellular growth factors activate membrane-located RTKs, which interact with farnesylated Ras GTPase proteins via the Grb2/SOS complex. Thereupon, Ras binds and activates Raf kinases, which undergo autophosphorylation upon homo- or heterodimerization and phosphorylate downstream MEK using ATP as a phosphate source. These MEKs, in turn, phosphorylate/activate MAPKs (e.g., JNK and p38 MAPK) and ERKs, which activate various oncogenic transcription factors (e.g., *c*-Myc, *c*-Jun, and *c*-Fos) involved in tumor progression and survival^[17,18]^. The gene transcription is regulated epigenetically by HDAC enzymes, among others^[10]^.

The central place of BRAF in this vital signaling pathway explains the oncogenic relevance of BRAF mutations. The most abundant mutation is BRAF^V600E^, which represents approximately 90% of all BRAF^V600^ mutations (which represent about 90% of all BRAF mutations) in melanoma and fosters tumor proliferation, survival, invasiveness, and drug resistance^[3]^. V600K (approximately 5%-6% of all BRAF^V600^-mutants), V600D, and V600R are further rare V600 mutations^[19,20]^. Mechanistically, the glutamate E600 drives the aggressiveness of BRAF^V600E^-mutant tumors since the glutamate anion is a phosphate-mimic leading to a permanent activation of the BRAF kinase activity also in the absence of activating stimuli from RTKs and Ras^[21]^. The V600 mutations form the class I BRAF mutations characteristic of Ras-independent active monomers, while non-V600 mutations creating activated BRAF homodimers independent from Ras belong to class II (e.g., G469A/V/R, L597Q/V, and K601E/N/T mutants), and non-V600 mutations (e.g., G466E/A/V, N581S/I/Y, D594G/N, G596D/R) leading to BRAF/CRAF heterodimers with increased Ras binding upon CRAF activation are classified as class III mutations [Figure 1]^[22]^. Notably, the rare non-V600 K601E mutation adjacent to the V600 site leads to activated BRAF^K601E^ dimers^[23]^. In addition, BRAF lysine K601 is acetylated by the HAT p300 and causes BRAF activation^[24]^. The intriguing regulation of oncogenic BRAF function by acetylation is discussed below.

#### BRAF inhibitors

Approximately 50% of cutaneous melanoma harbor activating BRAF mutants. Thus, the development of BRAF^V600E^ kinase inhibitors (e.g., vemurafenib, dabrafenib, and encorafenib) was a milestone in the therapy of V600-mutant melanoma^[7,25]^. Vemurafenib, approved by the FDA in 2011 for the therapy of metastatic melanoma, selectively binds to the ATP-binding site of BRAF^V600E^[26]^^. Dabrafenib, approved in 2013, exhibits higher binding affinity to BRAF^V600E^ compared to other BRAF inhibitors^[27]^. Encorafenib is an ATP-competitive Raf inhibitor approved for metastatic melanoma therapy, where it outperformed vemurafenib^[28]^. However, BRAF inhibitor resistance, as well as the low response of other non-melanoma cancers, e.g., BRAF-mutant CRC, limits the clinical application of these promising drugs^[7,29]^. The combination with MEK inhibitors or EGFR inhibitors appears to be suitable to prevent and overcome BRAF inhibitor resistance mechanisms, albeit with increased adverse effects^[30,31]^. Melanoma patients suffering from V600-mutant melanoma showed improved results upon combination therapy (BRAF inhibitor plus MEK inhibitor, e.g., vemurafenib plus cobimetinib, dabrafenib plus trametinib, and encorafenib plus binimetinib) compared with the corresponding BRAF inhibitor monotherapy^[32,33]^. Since the first generation of BRAF^V600E^ inhibitors is usually only weakly active against non-V600 BRAF-mutant cancers, compounds targeting BRAF-mutant dimers were developed (e.g., PLX8394 and lifirafenib), which showed promising activities against non-V600 BRAF-mutant lung cancers resistant to vemurafenib^[34,35]^. Unlike the clinically approved multi-kinase inhibitor sorafenib, which exclusively inhibits Raf dimers, lifirafenib is a pan-Raf inhibitor targeting both monomers and dimers. In addition, lifirafenib functions as an EGFR inhibitor and thus can also block bypassing resistance mechanisms. In contrast, PLX8394 acts as a “paradox breaker”. The effect of “paradoxical activation”, i.e., the BRAF^V600^-selective inhibitor-mediated promotion of the activating dimerization of wildtype Rafs and non-V600-mutant BRAF proteins, is well-documented and based on the (“in” or “out”) conformation changes of the regulatory αC-helix and catalytic DFG motifs of the ATP binding pocket upon inhibitor binding. Phosphorylation of the Raf activation loop leads to the Raf-activating CIDI conformation (αC-helix-in and DFG-in conformation) as a prerequisite of dimerization, while the BRAF inhibitors vemurafenib and dabrafenib bind to the ATP binding site leading to the CODI conformation (αC-helix-out and DFG-in). Sorafenib and pan-Raf inhibitors lead to the CIDO conformation (αC-helix-in and DFG-out), which can also promote paradoxical activation. However, the so-called paradox breakers can stabilize the “out”-position of the crucial R506 residue in the αC-helix, which disrupts the dimer interface of BRAF/BRAF and BRAF/CRAF dimers^[36,37]^.

### HDACs and their inhibitors

#### An overview of HDAC biology

**Figure 2:**
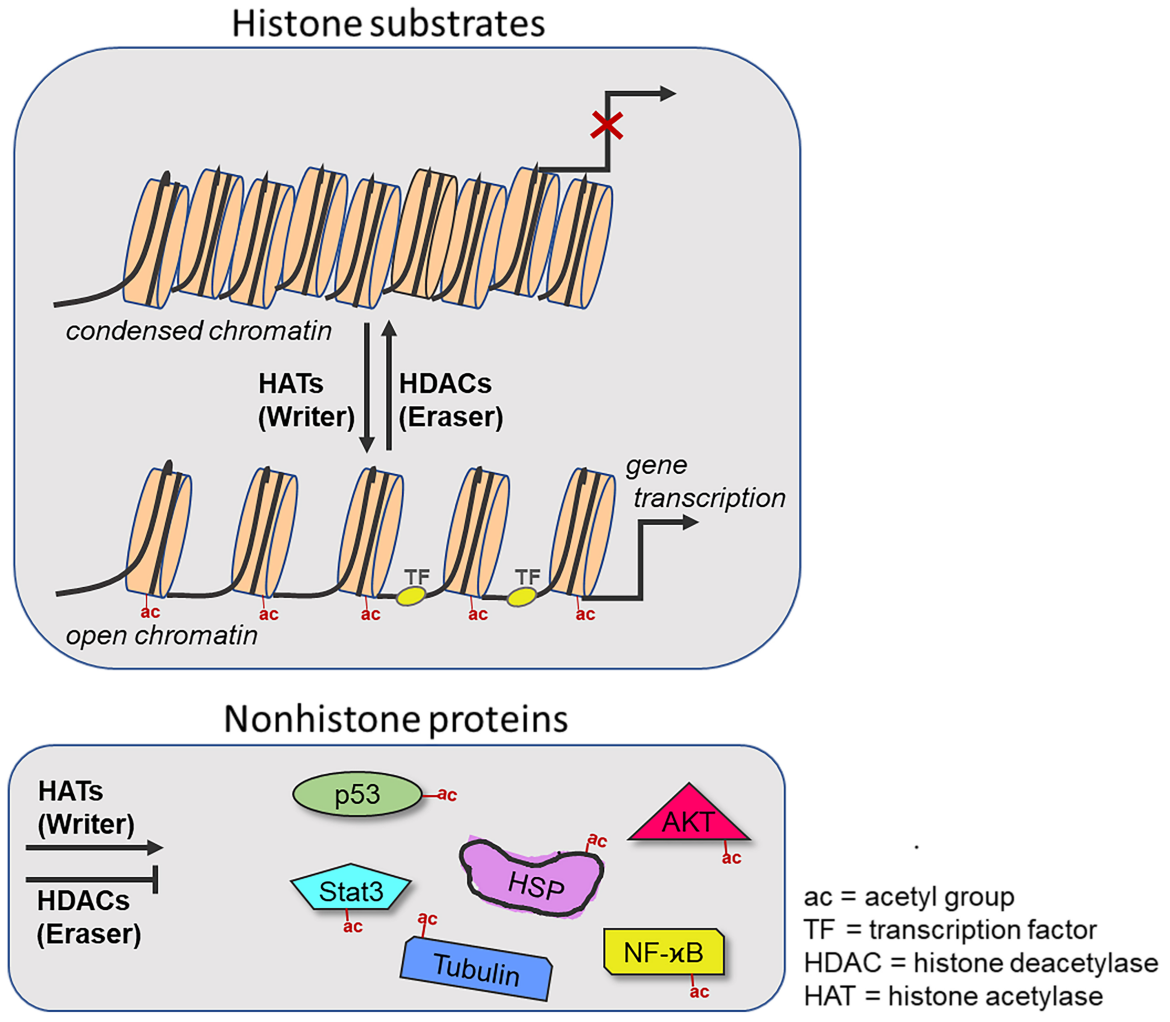
Histone modification of Histone acetylases and Histone deacetylases. Chromatin is in a closed conformation when acetylation groups are removed from histones by HDACs, thereby preventing access to key TF. Acetylases attach ac to histones, which contributes to a more open chromatin conformation that allows transcription factors to bind to DNA (bottom). HATs and HDACs also target numerous non-histone substrates, including transcription factors (e.g., p53), chaperones (e.g., Hsp90) and signaling molecules (e.g., NF-κB). The balance between the activities of HATs and HDACs serves as a critical regulatory mechanism for gene expression.

Histones (core histones H2A, H2B, H3, H4, and linker histone H1) are nuclear proteins that bind DNA to form the characteristic nucleosome structures of condensed chromatin^[38]^. The post-translational modification of histones includes methylation, phosphorylation, and acetylation processes, among others, which regulate the interaction of histones with DNA and other proteins. The acetylation of histone lysines by HATs (writers) using acetyl-CoA reduces the histone-DNA interaction, leading to decondensed chromatin, which is a prerequisite of gene transcription and DNA replication [Figure 2]^[39]^. Additionally, reader proteins (e.g., BET/bromodomain and extraderminal proteins) can recognize acetyl-histones to promote transcription and are promising anticancer drug targets^[40]^. In contrast, HDACs (erasers) remove the regulatory *N*-acetyl groups from lysine residues of target proteins, which include histones and several non-histone proteins. In terms of histone deacetylation, the positive charge of the affected histone increases based on the ammonium moieties of the deacetylated lysines, which enables a tighter histone-DNA binding and, consequently, chromatin condensation along with repression of gene transcription (especially of genes involved in apoptosis and cell cycle processes)^[41]^. Thus, HDACs play a crucial role in regulating the expression of oncogenes and tumor suppressor genes, especially since these vital enzymes are overexpressed in several cancers. Furthermore, HDACs target non-histone proteins that are involved in many vital processes in cancer cells and their microenvironment^[42]^.

HDACs are subdivided into four classes [Table 1]. Classes I (nuclear HDAC1-3, HDAC8), IIA (HDAC4, HDAC5, HDAC7, HDAC9, located in the nucleus or cytoplasm), IIB (cytoplasmic HDAC6 and HDAC10), and IV (HDAC11, located in the nucleus or cytoplasm) are Zn-dependent deacetylases with a catalytic Zn(II) ion in their active site. This important Zn(II) ion coordinates the catalytic water molecule required for acetamide hydrolysis. In contrast, class III HDACs are NAD^+^-dependent enzymes commonly known as sirtuins [Sir2-like proteins, seven human sirtuins (SIRT1-7), located in the nucleus or mitochondria, Table 1]^[41]^.

**Table 1:** Classification and localization of HDAC enzymes

**Class**	**HDACs**	**Localization**
I	HDAC1-3, HDAC8	Nucleus
IIA	HDAC4, HDAC5, HDAC7, HDAC9	Nucleus, cytoplasm
IIB	HDAC6, HDAC10	Cytoplasm
III	SIRT1-7	Nucleus (SIRT1, 2, 6, 7), cytoplasm (SIRT2), mitochondria (SIRT3-5)
IV	HDAC11	Nucleus, cytoplasm

HDAC: Histone deacetylase; SIRT1-7: seven human sirtuins.

#### Inhibitors of Zn-dependent HDACs

HDACs are overexpressed in melanomas and regulate genes involved in melanoma proliferation^[16]^. Based on the crucial role of Zn(II)-dependent HDACs in tumor development and progression and in line with their overexpression in various cancers, several HDAC inhibitors were identified and developed as anticancer drugs. The anticancer mechanisms of HDAC inhibitors include cell cycle arrest (via upregulation of p53 and p21), induction of apoptosis (intrinsic pathway via upregulation of p53 and pro-apoptotic Bax or Bak, and downregulation of anti-apoptotic Bcl-2 and Bcl-XL), and autophagy^[43]^. The bacterial metabolite butyrate was early described as an HDAC inhibitor, which is produced by intestinal microbiota and has an important role in intestinal homeostasis and prevention of gastrointestinal disorders including CRC^[44]^. The anti-epileptic drug valproate is a synthetic butyrate analog that also has HDAC inhibitory activity^[45]^. Further prominent natural HDAC inhibitors are the hydroxamic acid trichostatin A and the depsipeptide romidepsin, the latter of which has already been approved for the therapy of cutaneous T cell lymphoma. The class I and II HDAC inhibitor, trichostatin A, has often been applied for mechanistic investigations, and a transcriptomic study in BRAF-mutant melanoma cells revealed downregulation of transcripts involved in the promotion of the BRAF-ERK1/2 pathway (e.g., protein kinase C δ upstream of BRAF, and MYC proto-oncogene downstream of BRAF) upon treatment with trichostatin A^[46]^. However, the other HDAC inhibitors approved by the FDA (U.S. Food and Drug Administration) for lymphoma or myeloma therapy (vorinostat, belinostat, and panobinostat) are synthetic compounds with hydroxamic acid zinc binding groups (ZBG). These synthetic pan-HDAC inhibitors function as *N*-acetyl lysine mimics and have a modular pharmacophore structure based on the ZBG that is connected via a linker system with a suitable cap moiety^[41,47]^. In addition to hydroxamic acids, other ZBGs such as *ortho*-aminobenzamides are found in promising HDAC inhibitors (e.g., entinostat, mocetinostat) currently in clinical trials, and the subtype-specific (HDAC1-3 and HDAC10 inhibitor) benzamide tucidinostat (also known as chidamide) has already been locally approved in China and Japan for the therapy of relapsed/refractory (R/R) lymphomas and advanced breast cancer^[41,48]^. Subtype-specific HDAC inhibitors are expected to possess better pharmacological profiles than pan-HDAC inhibitors, as well as improved anticancer activities. Several HDAC6-selective inhibitors were disclosed, which possess immunomodulatory properties in various cancers, thus sensitizing tumors to immune therapy (e.g., checkpoint inhibitor therapy)^[15]^. Analogously, selective HDAC10 inhibitors or dual HDAC8/HDAC10 inhibitors have shown promising antitumor activities since HDAC10 contributes to drug resistance^[49]^.

HDAC inhibitor monotherapy has often revealed only meager clinical responses in patient studies, and solid tumors appeared to be relatively insensitive to HDAC inhibitor therapy^[13]^. Prostate cancer cells, for instance, undergo epithelial-to-mesenchymal transition (EMT) upon treatment with HDAC inhibitors^[50]^. However, the combination of HDAC inhibitors with other anticancer drugs (kinase inhibitors, hormone or immune therapy) has led to promising responses^[51]^. Chimeric drug candidates can also harbor dual and synergistic anticancer mechanisms. Since the cap moiety of classic HDAC inhibitors such as vorinostat can be used for chemical modifications without HDAC inhibition loss, numerous dual and multimodal HDAC inhibitors were designed, of which CUDC-101 (HDAC/EGFR/Her2 inhibitor) and CUDC-907 (also known as fimepinostat, HDAC/PI3K inhibitor) are the most prominent examples that have entered clinical trials^[52]^. Notably, dual HDAC/BRAF^V600E^ inhibitors based on the chemical structures of sorafenib and dabrafenib were meanwhile disclosed with higher antiproliferative activity against BRAF^V600E^-mutant cells (melanoma or CRC cells) compared with BRAF-wildtype cells^[52,53]^.

#### An overview of sirtuin biology

Sirtuins are NAD^+^-dependent HDACs involved in important cell biological processes, including cell death, metabolism, oxidative stress, and inflammation. Thus, they play a vital role in the development and progression of various human diseases including cancer. SIRT1-7 were described. The sirtuins can be distinguished by their cellular localization and their catalytic mechanisms. SIRT1, 2, 6, and 7 are located in the nucleus, while SIRT3-5 are found in the mitochondria. Mechanistically, SIRT1-3 and 7 are predominantly deacetylases, while SIRT4 and SIRT6 also have ADP-ribose transferase activities, and SIRT5 regulates various post-translational lysine modifications (e.g., malonylation, succinylation, and glutarylation)^[54]^. The sirtuin deacetylase functions by cleaving the glycosidic bond of the NAD^+^ co-factor upon acetyl lysine substrate binding followed by acetyl transfer forming *O*-acetyl-ADPR, nicotinamide, and deacetylated lysine^[55]^. The product nicotinamide can also function as a sirtuin feedback inhibitor (see below). Sirtuin substrates include various histone and non-histone proteins. For instance, acetylated K601 of BRAF proteins is a target of SIRT1^[24]^.

#### Sirtuin modulators

Notably, sirtuins can be regulated either positively or negatively by small-molecule activators and inhibitors, respectively. Resveratrol, a natural polyphenol of grapes, was described as an activator of SIRT1 in 2003. Synthetic compounds such as SRT2104 are more potent SIRT1 activators than resveratrol. Randomized controlled trials with resveratrol or SRT2104 showed SIRT1 activation in humans. However, there are also studies showing no effects of resveratrol or SRT2104 therapy^[54]^. In contrast to resveratrol, its close analog 4’-bromo-resveratrol (4’-BR) is an inhibitor of SIRT1 and other sirtuins such as SIRT3, while synthetic indole derivatives such as selisistat are selective SIRT1 inhibitors^[54,56]^. Selisistat entered clinical trials for the treatment of Huntington’s disease^[57]^. Nicotinamide is formed during the deacetylase reaction catalyzed by sirtuins, but it is also a SIRT1 and SIRT2 inhibitor (feedback inhibition). A phase 1 clinical study with R/R lymphoma patients receiving a combination of nicotinamide with the HDAC inhibitor vorinostat revealed promising results (response: 24%; stable disease: 57%)^[54,58]^.

## CELL DEATH AND SURVIVAL IN BRAF-MUTANT CANCERS

### Apoptosis in BRAF-mutant cancers

Apoptosis is a common form of programmed cell death induced by various stress factors including anticancer agents. The intrinsic apoptosis pathway is based on mitochondrial cytochrome c release and initiator caspase-9 activation, while the extrinsic pathway uses cell surface death receptors that activate initiator caspase 8. The tumor suppressor p53, also known as “guardian of the genome”, is an important upstream activator of cell cycle arrest and (intrinsic and extrinsic) apoptosis. It upregulates the expression of p21, PUMA, NOXA, Bak, APAF-1, TRAIL, and DR5. However, in various cancers, p53 is often downregulated to promote the proliferation and survival of tumor cells^[59,60]^. Activated MAPK signaling promotes cell growth and proliferation while inhibiting intrinsic apoptosis (i.e., apoptosis evasion). The Ras-MAPK-ERK pathway regulates pro-apoptotic (e.g., Bcl-2 and Bcl-XL) and anti-apoptotic factors (e.g., Bax and Bak forming a mitochondrial pore during apoptosis) of the Bcl-2 family, which control the permeability of the mitochondrial outer membrane. Increased permeability releases cytochrome c from the mitochondria, which leads to the formation of the apoptosome (together with initiator pro-caspase 9 and APAF-1) followed by activation of executioner caspases (e.g., caspase 3) and apoptosis induction. BRAF^V600E^ mutation prevents apoptosis induction by ERK-dependent downregulation of the pro-apoptotic Bad and Bim proteins, which are BH3-only members of the Bcl-2 family^[61]^. BH3-only proteins either neutralize anti-apoptotic Bcl-2, Bcl-XL, and Mcl-1, or activate Bax and Bak. Bad and Bim are potent activators of the Bax/Bak channel in the mitochondrial membrane^[62-64]^. Targeting Bcl-2 family proteins is a proven strategy to tackle apoptosis-resistant cancers, and the Bcl-2 inhibitor venetoclax is currently applied for the therapy of leukemias (CLL, ALL)^[60]^. In contrast to monotherapy with BH3-mimetics, the combination of Mcl-1 inhibitors with Bcl-2 inhibitors showed promising activities against BRAF^V600E^-mutant and BRAF-wildtype melanomas^[65]^. However, trametinib-resistant BRAF^V600^ melanomas with activated ERK1/2 were resistant to the Mcl-1 inhibitor S63845 because of ERK1/2-mediated upregulation of Mcl-1 and suppression of Bim, while cells with high Bim levels but low ERK1/2 and Mcl-1 activities were especially sensitive to Mcl-1 inhibition^[66]^. Thus, apoptosis-correlated factors are promising drug targets in BRAF-mutant cancers, but can also have limited activities in BRAF/MEK inhibitor-resistant cancers requiring further actions to eliminate resistant cells.

BRAF^V600E^ melanoma cells are killed by vemurafenib via inhibition of MAPK signaling followed by apoptosis induction; however, in patients treated with vemurafenib, resistance usually develops after 6-8 months on average^[8]^. The targeting of MAPK signaling by BRAF and other Raf inhibitors can reactivate ERK1/2 via CRAF homodimers or BRAF/CRAF heterodimers, leading to the upregulation of the anti-apoptotic Mcl-1 protein. PLX4720-mediated apoptosis resistance via upregulated Mcl-1 was accompanied by highly invasive properties in NRAS-mutant melanoma cells^[67]^. RTKs such as EGFR, VEGFR, PDGFR/KIT, *c*-Met, FGFR, and IGF-1R are located upstream of Ras-MAPK signaling, which was associated with MAPK reactivation and bypassing mechanisms, leading to BRAF-mutant cell survival and the prevention of cell death. RTKs also upregulate PI3K-AKT signaling, which plays a crucial role in bypassing the effects of MAPK inhibitors^[6]^. The heat shock protein Hsp90 is another important factor for apoptosis resistance in BRAF-mutant cancers since it stabilizes numerous oncogenes including Raf kinases, such as CRAF. Hsp90 inhibition led to apoptosis induction in resistant BRAF-mutant cancer cells by reducing the levels of PDGFRβ, IGF-1R, AKT and Mcl-1 while upregulating Bim. Further factors involved in apoptosis resistance of BRAF-mutant cancers include hedgehog signaling (GLI1/2), p90RSK, AMPK, and various epigenetic factors such as histone demethylases, DNMTs (DNA methyltransferases), and HDACs^[8]^.

### Autophagy and senescence in BRAF-mutant cancers

Autophagy (also known as macroautophagy) is a catabolic process that maintains cell homeostasis through lysosomal degradation of damaged organelles and protein aggregates in response to stress factors. Under normal conditions, the initiating factors ULK1 and ATG13 are inhibited by mTOR; however, upon autophagy initiation, the inhibition by mTOR is prevented and the ULK complex is formed (by ULK1, FIP200, and ATGs 13 and 101). The ULK complex builds the PI3K complex with other proteins such as Beclin 1 (also known as Atg6). Phosphorylation of Beclin 1 by ULK1 leads to the recruitment of additional proteins, which contribute to the formation of the autophagosome. Expansion of the phagophore includes processing of LC3 by cleavage to LC3-I (by cysteine protease ATG4B) and conjugation to phosphatidylethanolamine (via ubiquitination-like processes involving ATGs 3 and 7, and the ATG12-ATG5-ATG16L1 complex). LC3-II and p62 interact in the autophagosome membrane and recognize ubiquitinated substrates. Finally, the autophagosomes interact with lysosomes, forming autophagolysosomes where the substrates are degraded^[68]^. Notably, autophagy is upregulated in BRAF-mutant melanoma, promotes BRAF^V600E^ melanoma survival, and conveys adaptive resistance to BRAF and MEK inhibitor therapy^[69-73]^. However, autophagy is a double-edged sword, playing either a pro-tumor or tumor-suppressive role in BRAF-mutant cancers. Pro-tumor autophagy comprises processes that downregulate apoptosis/cell death in favor of autophagy. AXL and MERTK are involved in apoptosis resistance to BRAF inhibitors in BRAF-mutant cells by promoting autophagy^[74,75]^. Further factors promoting pro-tumor autophagy and drug resistance include AMPK, ER stress factors (GRP78, JNK), the transcription factor TFEB (transcription factor EB), and sirtuins^[68]^.

The tumor-suppressive role of autophagy is associated with senescence. Senescence is a robust and enduring cell cycle arrest connected with cell growth inhibition but also with resistance to cell death mechanisms induced by anticancer drugs. Encorafenib induced autophagy and senescence in BRAF^V600E^ melanoma cells, while the combination of encorafenib with an autophagy inhibitor showed increased antiproliferative activity against BRAF-mutant cells^[76]^. Heterogeneous senescence mechanisms can be induced in BRAF-mutant cells with the MITF/miR-579-3p axis as a regulator upon treatment with MAPK inhibitors^[77]^. Moreover, vemurafenib selectively induced robust non-canonical senescence in BRAF^V600E^ melanoma cells accompanied by the release of cytokines from treated cells^[78]^.

## HDACS AND CELL DEATH IN RESISTANT BRAF-MUTANT CANCERS

### HDACs and apoptosis

**Figure 3:**
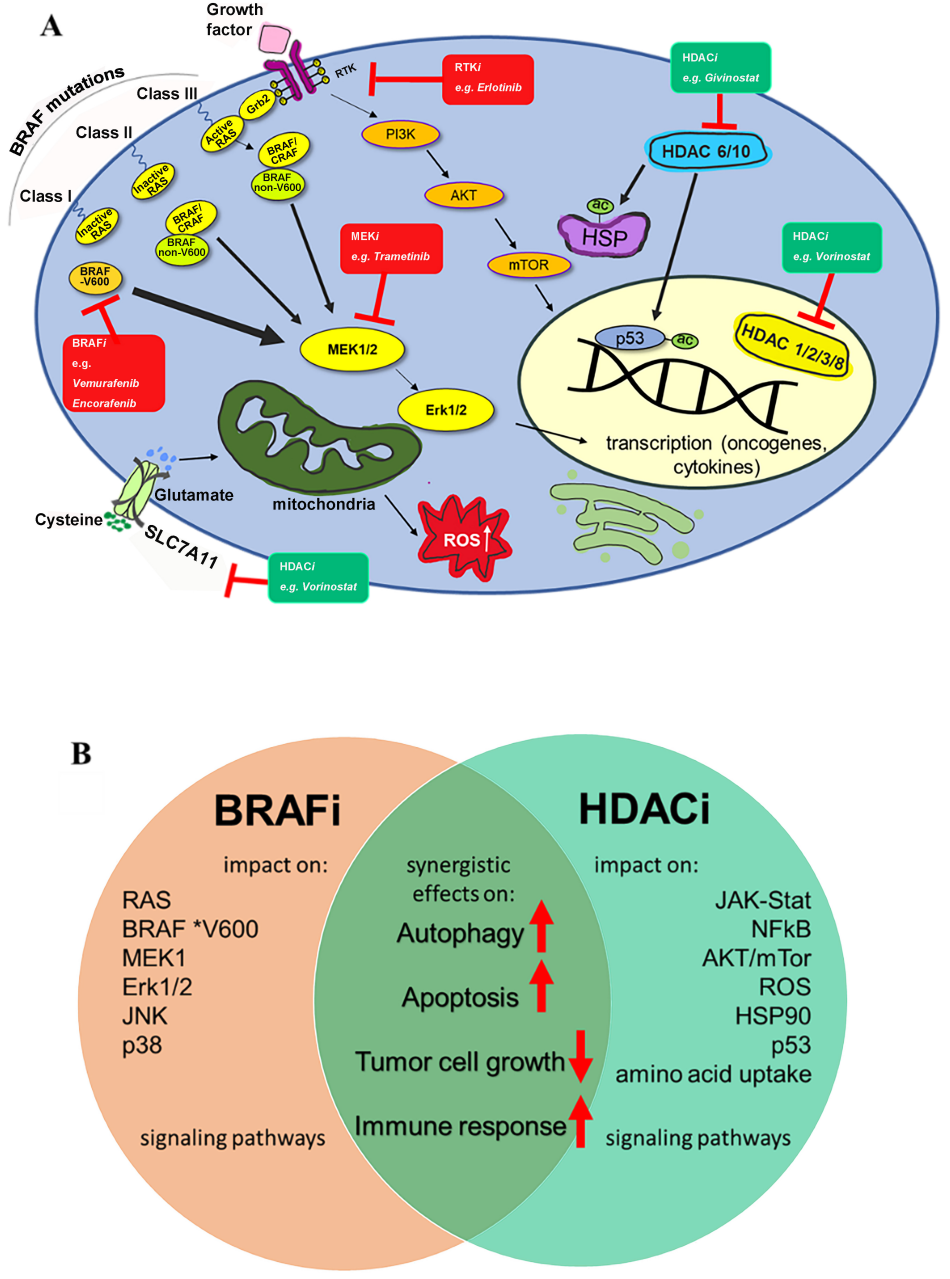
(A) Schematic representation of cellular targets of HDACi and targeted BRAF mutant therapy concepts of relevance for cell death mechanisms. Classification of BRAF mutations is depicted according to Ras dependency. Class I BRAFi like vemurafenib, a reversible ATP competitive inhibitor of the BRAF serine-threonine kinase, act as a repressor of the activated MAPK signaling pathway. Class II and Class III inhibitors like trametinib are downstream inhibitors of the MAPK pathway, the MEK or inhibitors of RTK. HDAC inhibitors targeting cancer cell-related pathways are described as a promising approach to overcoming BRAF mutation-induced therapy resistance. The cellular HDAC-related targets are located either in the cytosol or the nucleus. For example, the inhibition of the cytosolic HDAC6/10 by the pan HDAC inhibitor givinostat leads to activation and acetylation of p53 together with disruption of Hsp90 chaperone function, leading to pronounced anticancer effects such as apoptosis and cell cycle arrest; (B) Synergistic effects of BRAF and HDAC inhibitors on cell death mechanisms. HDACi: Histone deacetylase inhibitors; BRAFi: BRAF inhibitors; BRAF: B-Raf, B-rapidly accelerated fibrosarcoma; MEK: MAPK/ERK kinase; RTK: receptor tyrosine kinases; ac: acetyl group; MAPK: mitogen-activated protein kinase.

HDACs are critically involved in cell survival processes, suppress apoptosis induction, and are correlated with BRAF inhibitor resistance in melanoma and other BRAF-mutant cancers [Figure 3]^[16,79]^. Thus, a detailed understanding of how HDACs mediate drug resistance is necessary to develop more efficient therapies for recurrent cancers. Depending on the cell conditions, HDACs promote the proliferation or survival of tumor cells, and thus, HDAC inhibitors can induce cell cycle arrest or cell death such as apoptosis.

The HDAC1-3 inhibitor entinostat, in combination with BRAF/MEK inhibitors, synergistically increased apoptosis induction in BRAF-mutant melanoma cells, thus killing cells that would have survived BRAF/MEK inhibitors alone. HDAC3 inhibition was responsible for the anticancer effects of entinostat. The combination of dabrafenib/trametinib with entinostat also exhibited increased tumor growth inhibition of A375 xenografts (regression of 70%), which was much higher than the regression observed for dabrafenib/trametinib (32%). It was shown that MGMT (O6-methylguanine-DNA methyltransferase) expression was a marker for sensitivity to entinostat/dabrafenib/trametinib therapy, and that this combination causes DNA damage, suppresses DNA repair genes, and downregulates HR (homologous recombination) and NHEJ (non-homologous end-joining) DNA repair mechanisms [Table 2]^[80]^.

**Table 2:** HDAC inhibitors and apoptosis in BRAF-mutant cancers

**HDAC**	**Inhibitor**	**Activity**
HDAC1-3	Entinostat	Enhanced apoptosis induction in BRAF-mutant melanoma cells in combination with dabrafenib/trametinib, DNA damage, suppression of DNA repair genes, downregulation of HR and NHEJ DNA repair, MGMT expression as a sensitivity marker, increased tumor growth inhibition of A375 xenografts (regression of 70%)
Class I and II HDACs	Givinostat	Apoptosis induction via p53 activation and reduction of BRAF expression in BRAF^V600E^ A375 melanoma
Pan-HDAC	Vorinostat	Activation of bypassing mechanisms upon exposure of BRAF^V600E^ A375 melanoma to PLX4720, sensitization to RTK and HDAC inhibitors (erlotinib, imatinib, and vorinostat), reduced cell survival, suppressed RB phosphorylation, increased apoptosis induction
Pan-HDAC	Vorinostat, belinostat, panobinostat	Downregulation of *SLC7A11* (cysteine-glutamate antiporter xCT gene), increased cytotoxic ROS levels in drug-resistant melanoma associated with apoptosis, *in vivo* activity in vemurafenib-resistant A375 melanoma clones, clinical activity in patients with advanced BRAF + MEK inhibitor-resistant melanoma
Pan-HDAC	Vorinostat	Re-established BIM-XL expression, increased apoptosis in the presence of PLX4720 and selumetinib, in BRAF inhibitor-resistant melanoma
Pan-HDAC	Panobinostat	Restored sensitivity in vemurafenib-resistant BRAF-mutant melanoma, suppression of PI3K signaling and *c*-Myc, induction of pro-apoptotic BIM and NOXA
Pan-HDAC	Romidepsin	Restored BIK expression and sensitivity in BRAF^V600E^-mutant M14 melanoma cell clones resistant to vemurafenib and trametinib *in vitro* and *in vivo*
Pan-HDAC	Panobinostat	Synergistic caspase-dependent apoptosis in combination with BET inhibitor (I-BET151) in patient-derived vemurafenib-resistant BRAF^V600E^ melanoma cells, induction of pro-apoptotic BIM and suppression of anti-apoptotic Bcl-2, Bcl-XL, and XIAP, inhibition of YAP1 and AKT, prolonged survival associated with tumor growth inhibition in mice with patient-1-post BRAF^V600E^ melanomas
HDAC8	PCI-34051	Restored apoptosis induction in vemurafenib-resistant cells, suppression of S897-EphA2 and AKT signaling, combination with PLX4720 inhibits treatment-naïve 1205Lu melanoma growth
Pan-HDAC	Romidepsin	Increased expression of Rap1, apoptosis induction and suppression of ERK1/2 in BRAF^V599E^-mutant melanoma
Pan-HDAC	Vorinostat	Apoptosis induction via caspase-8 in BRAF^V600E^ CRC by suppression of basal and selumetinib-induced expression of *c*-FLIP_L_
HDAC1-3	Entinostat	Caspase-8-dependent apoptosis induction in BRAF^V600E^ CRC by suppression of basal and selumetinib-induced expression of *c*-FLIP_L_, combination with selumetinib inhibits BRAF^V600E^ HT-29 CRC xenograft growth via increased H4 acetylation and reduced STAT3 activation, apoptosis via caspase-3 activation
Pan-HDAC	Vorinostat	DNMT1 degradation, reduced cell viability both in sensitive and in vemurafenib-resistant BRAF-mutant melanoma cells

HDAC: Histone deacetylase; RTK: receptor tyrosine kinases; ac: acetyl group; CRC: colorectal cancer; MEK: MAPK/ERK kinase; BRAF: B-Raf, B-rapidly accelerated fibrosarcoma.

The “guardian of the genome” p53 is an important tumor suppressor involved in the induction of apoptosis and cell cycle arrest upon excessive cell damage. The acetylated p53 protein is a target of HDACs (e.g., HDAC1 and HDAC6), which inactivate p53 by deacetylation^[81,82]^. HDAC inhibition by givinostat activated wildtype p53 in A375 BRAF-mutant melanoma cells, leading to apoptosis induction and reduction of BRAF expression, while BRAF-mutant SK-MEL-28 cells with mutant oncogenic p53 were less sensitive to givinostat-mediated apoptosis [Table 2 and Figure 3]^[83]^. Thus, the identification of the p53 mutation status in BRAF-mutant melanoma patients can be relevant to the clinical outcome of HDAC inhibitor therapy. It was shown that HDAC2 downregulates p53 in celastrol-resistant Malme3M melanoma cells, leading to apoptosis resistance^[84]^. In BRAF-mutant SK-MEL-3 melanoma cells, the natural HDAC inhibitor trichostatin A downregulated pro-apoptotic p53 and anti-apoptotic Bcl-2 at low doses in line with a favored cell cycle arrest instead of apoptosis induction. Only higher doses of trichostatin A above the concentrations required for HDAC inhibition led to apoptosis induction by this compound in these cells^[46]^.

Acquired cancer resistance can be accompanied by new drug vulnerabilities^[85]^. BRAF^V600E^ A375 melanoma cells are initially highly sensitive to the vemurafenib analog PLX4720, but insensitive to targeted and epigenetic therapies, including HDAC inhibition. Prolonged systemic exposure of these BRAF-mutant cells to PLX4720 activated bypassing mechanisms mediated by RTKs and HDACs, which sensitized the cells to RTK and HDAC inhibitors (erlotinib, imatinib, and vorinostat), leading to reduced cell survival [Figure 3]. Sensitization of cells to vorinostat by PLX4720 was associated with suppressed RB phosphorylation, leading to increased apoptosis induction [Table 2]^[86]^.

BRAF^V600E^ melanoma resistant to BRAF and MEK inhibitors exhibited increased levels of reactive oxygen species (ROS), which drive MAPK-signaling reactivation. Treatment of resistant melanoma cells with the pan-HDAC inhibitor vorinostat downregulated *SLC7A11* (the gene of the cysteine-glutamate antiporter xCT), which further increased intracellular ROS levels to a cytotoxic level specifically in the drug-resistant cells, triggering apoptosis [Table 2 and Figure 3]. These findings were confirmed *in vivo* using four vemurafenib-resistant A375 melanoma clones with elevated p-MEK levels (harboring BRAF amplification, NRAS^Q61K^, or increased TGF-β signaling), which were sensitive to treatment with vorinostat, belinostat, and panobinostat. Thus, drug-resistant BRAF-mutant melanoma developed a specific sensitivity to HDAC inhibitor therapy, which was also clinically verified. Three patients with advanced BRAF+MEK inhibitor-resistant melanoma, who suffered from progression upon dabrafenib plus trametinib therapy, were treated with vorinostat (360 mg/day, orally), which suppressed *SLC7A11* in taken biopsies. In addition, vorinostat eradicated tumor cells with MAPK inhibitor resistance mutations such as KRAS^G12C^ and NRAS^Q61H^ in the biopsy samples^[87]^. A more recent clinical study with 22 BRAF+MEK inhibitor-resistant BRAF^V600^-mutant melanoma patients revealed that the daily oral dose of 360 mg vorinostat was well-tolerated and showed an overall response rate of 9%, including one complete response. Drug-inactivating mutations in MEK and NRAS had appeared in eight patients and were eliminated by vorinostat therapy in three of them^[88]^.

Acquired resistance of BRAF-mutant melanoma cells to vemurafenib and PLX4720 was accompanied by ERK1/2 reactivation followed by repression of the pro-apoptotic proteins BIM-XL and Bmf. While MEK inhibitors could not induce apoptosis in BRAF inhibitor-resistant cells, the HDAC inhibitor vorinostat was able to re-establish BIM-XL expression and to increase cell death in the presence of PLX4720 and selumetinib [Table 2]^[89]^. Panobinostat restored BRAF inhibitor sensitivity in BRAF-mutant melanoma cells with acquired vemurafenib resistance by suppressing PI3K signaling and *c*-Myc, which was accompanied by the induction of pro-apoptotic BIM and NOXA [Table 2]^[90]^. Trichostatin A also suppressed *c*-Myc in BRAF-mutant SK-MEL-3 melanoma cells; however, this compound predominantly induced mitotic arrest and cytostatic effects, rather than causing cell death^[84]^. Pro-apoptotic BIK expression mediated apoptosis induction in BRAF^V600E^-mutant M14 melanoma cell clones responding to vemurafenib and trametinib therapy. Clones that are resistant to apoptosis induction by BRAF and MEK inhibition responded only via cell cycle arrest and exhibited downregulated BIK. However, HDAC inhibition by treatment with romidepsin restored BIK expression as well as cell sensitivity to vemurafenib plus trametinib treatment. The beneficial BIK-restoring effect of romidepsin in combination with vemurafenib and trametinib was also shown in xeno-transplanted M14 tumors with low BIK levels *in vivo* [Table 2]^[91]^.

The pro-apoptotic effects of HDAC inhibition can be boosted by inactivation of reader proteins. The combination of the HDAC inhibitor panobinostat with the BET inhibitor I-BET151 showed synergistic caspase-dependent apoptosis induction in patient-derived BRAF^V600E^ melanoma cells (patient-1-post, patient-3-post) with acquired vemurafenib resistance, but not in melanocytes [Table 2]. Notably, the resistant melanoma cells were also resistant to monotherapy with panobinostat or I-BET151. Synergistic panobinostat plus I-BET151 activated pro-apoptotic BIM and suppressed anti-apoptotic Bcl-2, Bcl-XL, and XIAP. Activating phosphorylation of YAP1 and AKT was also inhibited, indicating downregulation of Hippo and AKT signaling pathways by combined HDAC and BET inhibition. Mice bearing patient-1-post BRAF^V600E^ melanomas were treated with panobinostat (4 mg/kg/day, i.p.) plus I-BET151 (15 mg/kg on 5 days per week, orally) and showed prolonged survival associated with tumor growth inhibition^[92]^. Notably, AKT was activated by HDAC8 in BRAF-mutant HT-29 CRC cells, leading to resistance to MEK1/2-ERK inhibitors^[93]^. HDAC8 expression is upregulated in resistant BRAF-mutant melanoma cells, where HDAC8 deacetylates c-Jun to reactivate MAPK signaling. HDAC8 mediated resistance by upregulating c-Jun and MAPK signaling via activation of RTKs such as EGFR, FGFR, and c-Met. The pan-HDAC inhibitor panobinostat and the selective HDAC8 inhibitor PCI-34051 restored apoptosis induction by vemurafenib in resistant cells by suppressing S897-EphA2 and AKT signaling, and the combination of PCI-34051 with PLX4720 inhibited the growth of treatment-naïve 1205Lu melanoma xenografts [Table 2]^[94]^. Notably, increased levels of HDAC8 in the cytoplasm of patients with metastatic BRAF-mutant melanoma were correlated with better survival, and the HDAC8 localization within tumor cells seems to be important for its biological effects, either positive or negative, on the cancer patient^[95]^.

The GTPase Rap1 is a regulator of Ras-MAPK signaling, which is overexpressed in BRAF^V599E^-mutant melanoma cells and forms a complex with BRAF^V599E^. The HDAC inhibitor romidepsin increased the expression of Rap1, leading to apoptosis induction and suppression of ERK1/2 phosphorylation in melanoma cells [Table 2]^[96]^.

Resistance to MEK1/2 inhibition in BRAF^V600E^ CRC was associated with upregulated STAT3 and its target *c*-FLIP_L_ via *c*-Met activation. Although MEK1/2 inhibition by trametinib and selumetinib reduced BRAF-mutant CRC cell viability, the induction of apoptosis was hampered by STAT3 and anti-apoptotic *c*-FLIP_L_. Basal and selumetinib-induced expression of *c*-FLIP_L_ was downregulated in BRAF-mutant CRC by treatment with the pan-HDAC inhibitor vorinostat, leading to caspase-8-dependent apoptosis induction [Table 2]. Analogously to the effects observed for vorinostat, basal and selumetinib-induced expression of *c*-FLIP_L_ was downregulated in BRAF-mutant CRC by treatment with the class I selective HDAC1-3 inhibitor entinostat, leading to caspase-8 dependent apoptosis induction. The combination of entinostat with selumetinib inhibited BRAF^V600E^ HT-29 CRC xenograft growth, which was accompanied by increased H4 acetylation, reduced STAT3 activation, and apoptosis induction via caspase-3 activation [Table 2]^[97]^. It is conceivable that subtype-specific entinostat and mocetinostat will have reduced adverse and off-target effects compared to the pan-HDAC inhibitor vorinostat in clinical studies.

HDAC inhibition promotes acetylation of the DNA methyltransferase DNMT1, leading to DNMT1 degradation^[98]^. The suppression of DNMT1 reversed interferon-mediated apoptosis resistance in melanoma cells^[99]^. BRAF-mutant melanoma with acquired resistance to vemurafenib showed increased levels of DNMT1, which is an eminent epigenetic factor in melanoma progression. DNMT1 degradation by vorinostat treatment reduced cell viability both in sensitive and vemurafenib-resistant melanoma cells [Table 2]^[100]^.

Hence, pan-HDAC inhibition can affect and impair tumor cell survival through the promotion of apoptosis in various BRAF-mutant tumor models. More precisely, class I HDACs (HDAC1-3) and HDAC8 play a crucial role in the prevention of apoptosis in BRAF-mutant cancers, including BRAF inhibitor-resistant cells, which can be targeted by isoenzyme-selective inhibitor molecules.

### HDACs and non-apoptotic cell death mechanisms, senescence and immunomodulation

Apoptosis is not the only way HDAC inhibitors kill melanoma cells, and HDAC inhibition can also bypass canonical cell death mechanisms. ROS formation, suppression of anti-apoptotic proteins Bcl-XL and Mcl-1, increased Beclin 1 and ATG5 levels, and promotion of autophagosome formation were discovered in BRAF^V600E^ patient-derived xenograft (PDX) melanoma cells treated with the ERBB and MAPK4 inhibitor neratinib, and synergy effects were observed in combination with HDAC inhibitors^[101]^.

Although the combination of vorinostat with the BRAF inhibitor PLX4720 activated caspase-dependent apoptosis in BRAF^V600E^ melanoma, caspase inhibition did not prevent necrotic cell death. Notably, necrosis by this combination therapy was neither prevented by the necrosis inhibitor necrostatin-1, an inhibitor of RIPK1 (receptor-interacting protein kinase 1), nor by knockdown of RIPK3 (receptor-interacting protein kinase 3), which indicates that RIPK1 and RIPK3 are not involved in HDAC-inhibitor-mediated necrosis [Table 3]. Combined vorinostat and vemurafenib also inhibited tumor growth of BRAF^V600E^ melanoma xenografts independent from caspase-3 activity^[102]^.

**Table 3:** HDACs and their effects on non-apoptotic cell death, senescence and immune therapy in BRAF-mutant cancers

**HDAC**	**Inhibitor**	**Activity**
Pan-HDAC	Vorinostat	Necrotic cell death in combination with PLX4720 in BRAF^V600E^ melanoma independent from RIPK1 and RIPK3, tumor growth inhibition of BRAF^V600E^ melanoma xenografts independent from caspase-3 activity in combination with vemurafenib
Pan-HDAC	Valproic acid	Synergistic antiproliferative activity against BRAF-mutant ARO thyroid cancer cells in combination with Ras inhibitor FTS, mitotic crisis by suppression of Ras, survivin, and aurora A
Pan-HDAC	AR42	AIF-mediated necroptosis and toxic autolysosome formation in dabrafenib/trametinib-resistant melanoma cells in combination with pazopanib, DNA damage, ATM and AMPK activation, inactivation of Hsp90, Hsp70, and GRP78 and suppression of Raf proteins (RAF-1, BRAF), anti-apoptotic proteins (Bcl-XL, Mcl-1), ERK1/2, AKT, and HDACs, prolonged survival of mice with BRAF/MEK inhibitor-resistant melanoma in combination with pazopanib
Pan-HDAC	GZ17-6.02	Suppression of HDACs in vemurafenib-resistant PDX BRAF^V600E^ melanoma cells, improved cell killing in combination with dabrafenib and trametinib, including non-apoptotic mechanisms, inactivation of AKT, mTOR, and MEK1/2-ERK1/2, activation of CD95 death receptor and autophagy, dysfunctional mitochondria leading to cell death
Pan-HDAC	4-phenyl-butyrate	Upregulation of MC1R in BRAF-mutant melanoma cells in combination with vemurafenib, increased response of BRAF^V600E^ A2058 melanoma to radiolabeled peptide [^212^Pb]DOTA-MC1L
Pan-HDAC	Panobinostat, vorinostat	Re-differentiation of BRAF^V600E^ PTC cells associated with increased radioiodine uptake and cytotoxicity, increased NIS expression by high histone acetylation of the *NIS* gene promoter, combination with MAPK inhibitors enhanced re-differentiation in BRAF^V600E^ cells
Pan-HDAC, class I and IV HDACs	Vorinostat, panobinostat, sodium butyrate, mocetinostat	Elimination of senescent SUR cells upon vemurafenib therapy (resistant to EZH2 and DNMT, class II HDAC and selective HDAC6 inhibitors) via induction of apoptosis and cell growth inhibition
Pan-HDAC	Quisinostat	High activities in combination with flavopiridol against cutaneous BRAF^V600E^ melanoma with acquired BRAF inhibitor resistance (MM249-R and SK-MEL28-R cell lines)
HDAC1-3/LSD1	Domatinostat	Elimination of senescent PDX BRAF-mutant MM27 melanoma cells, inhibition of MM27 xenograft growth in combination with USP7 inhibitor P5091, apoptosis induction, reduced senescent cell numbers
Pan-HDAC, HDAC1-3	Panobinostat, entinostat, mocetinostat	Enhanced immune therapy by induction of PD-L1 expression BRAF-mutant (WM983A, WM793), upregulated histone acetylation in the PD-L1 gene, chromatin relaxation, prolonged survival of B16F10-bearing mice in combination with PD-1 antibody
Pan-HDAC	Sodium butyrate	Re-established NK cell recognition in the presence of vemurafenib, induction of MICA and nectin-2
Pan-HDAC	Valproic acid, vorinostat	G2/M cell cycle arrest in BRAF^V600E^ PF49 ATC cells, upregulated expression of PD-L1
Pan-HDAC	GZ17-6.02	Upregulated class I MHCA and suppressed PD-L1 in PDX BRAF^V600E^ melanoma cells, downregulation of HDACs (HDAC3, HDAC5, HDAC6, HDAC7, and HDAC8)
HDAC6	Tubastatin A, nexturastat A, suprastat, SS208, KS2507	Augmented antitumor immunity in BRAF-mutant melanoma (WM983A, WM793, SM1), upregulated expression of MHCI and melanoma antigens (gp100, MART1, TYRP1, and TYRP2), suppression of PD-L1 and M2-polarized macrophages

HDAC: Histone deacetylase; RTK: receptor tyrosine kinases; ac: acetyl group; Ras: rat sarcoma; FTS: farnesylthiosalicylic acid; MEK: MAPK/ERK kinase; PDX: patient-derived xenograft; MAPK: mitogen-activated protein kinase; BRAF: B-Raf, B-rapidly accelerated fibrosarcoma; RIPK1: receptor-interacting protein kinase 1; RIPK3: receptor-interacting protein kinase 3.

Valproic acid in combination with the Ras inhibitor farnesylthiosalicylic acid (FTS) showed synergistic antiproliferative activity against BRAF-mutant ARO thyroid cancer cells, which was accompanied by suppression of Ras, survivin, and aurora A, leading to a mitotic crisis [Table 3]^[103]^.

The combination of the HDAC inhibitor AR42 with the multi-kinase inhibitor pazopanib efficiently killed dabrafenib/trametinib-resistant melanoma cells by AIF-mediated necroptosis and toxic autolysosome formation. AR42 induced DNA damage followed by ATM and AMPK activation, and inactivated the heat shock proteins Hsp90, Hsp70, and GRP78, leading to the suppression of Raf proteins (RAF-1, BRAF), anti-apoptotic proteins (Bcl-XL, Mcl-1), ERK1/2, AKT, and various HDACs (e.g., HDAC6). AR42 plus pazopanib also distinctly prolonged the survival of mice with BRAF/MEK inhibitor-resistant melanoma [Table 3]^[104]^.

The drug GZ17-6.02 (a combination of 10% curcumin, 13% harmine, and 77% isovanillin) suppressed the expression of various HDACs in vemurafenib-resistant PDX BRAF^V600E^ melanoma cells, and showed improved cell killing in combination with dabrafenib and trametinib by apoptotic and non-apoptotic mechanisms. Together with the inactivation of AKT, mTOR, and MEK1/2-ERK1/2, activation of the CD95 death receptor and autophagy caused dysfunctional mitochondria, leading to pronounced melanoma cell death [Table 3]^[105]^.

HDAC10 maintained BRAF inhibitor resistance and proliferation of BRAF^V600E^ A375 and WM793 melanoma cells by suppressing the glycoprotein SPARC (secreted protein acidic and rich in cysteine) via regulation of histone H3 acetylation in the *SPARC* gene regulatory elements together with the HAT p300. Depletion of HDAC10 upregulated SPARC expression and re-sensitized resistant cells to vemurafenib via AMPK activation and autophagy induction^[106]^.

Radiotherapy is often applied for the therapy of melanomas, and radiotherapy-induced cell death mostly comprises mitotic catastrophe upon DNA damage. Other radiotherapy-mediated cell death mechanisms include apoptosis and autophagy, while senescence can also be induced as a survival mechanism^[107]^. Radiolabeled peptides as radiopharmaceuticals targeting the melanocortin-1 receptor (MC1R) can selectively attack and eliminate melanomas^[108]^. Combined inhibition of BRAF (by vemurafenib) and HDAC (by 4-phenylbutyrate) upregulated MC1R expression in BRAF-mutant melanoma cells leading to an increased response of BRAF^V600E^ A2058 melanoma-bearing mice to the MC1R-targeting radiolabeled peptide [^212^Pb]DOTA-MC1L [Table 3]^[109]^. However, there is also evidence that upregulated MC1R expression in melanomas leads to immune evasion and reduced T cell response by repression of CXCL9-11^[110]^.

Radioiodine therapy is applied for the treatment of differentiated thyroid cancer (DTC), including papillary thyroid cancer (PTC), due to the improved uptake of DTC cells based on the expression of the sodium iodide symporter (NIS). Radioiodine refractory DTC is associated with a poorer prognosis and develops as a consequence of a cell dedifferentiation process leading to the suppression of NIS and other iodine-metabolizing factors. Panobinostat was able to induce a re-differentiation of PTC cells associated with increased radioiodine uptake and cytotoxicity both in BRAF^V600E^ and BRAF-wildtype cells, while dabrafenib or selumetinib only exerted a re-differentiation in BRAF^V600E^ cells. The increased NIS expression is based on the higher histone acetylation level at the *NIS* gene promoter. Notably, the combination of panobinostat with MAPK inhibitors led to enhanced re-differentiation in BRAF^V600E^ cells compared with HDAC inhibition alone^[111]^. Similar effects (upregulated NIS and increased radioiodine uptake) were also observed for the combination of vorinostat with vemurafenib in BRAF^V600E^ thyroid cancer cells [Table 3]^[112]^.

HDACs apparently also play an eminent role in the senescence of BRAF^V600E^ melanoma cells, which is a form of acquired resistance to long-term vemurafenib treatment, leading to expression changes of various epigenetic factors such as EZH2 (downregulation), DNMT3b (downregulated), and HDACs (upregulation). The pan-HDAC inhibitors vorinostat, panobinostat, and sodium butyrate, as well as mocetinostat (a class I- and IV-specific HDAC inhibitor), were able to eliminate senescent cells that have survived vemurafenib therapy (which were dubbed SUR cells) through induction of apoptosis and cell growth inhibition [Table 3]. In line with the expression results of other epigenetic factors, SUR cells were resistant to EZH2 and DNMT inhibitors. Additionally, class II HDAC inhibitors and selective HDAC6 inhibitors were also ineffective against SUR cells^[113]^.

Since a CDK (cyclind-dependent kinase) inhibitor also showed activity against SUR cells, the combination of HDAC inhibition with CDK inhibition appears to be promising in terms of concomitant apoptosis induction and cell cycle arrest in resistant BRAF-mutant cancers. Quisinostat (pan-HDAC inhibitor) plus flavopiridol (CDK inhibitor) showed high activities against cutaneous BRAF^V600E^ melanoma cells with acquired BRAF inhibitor resistance (MM249-R and SK-MEL28-R cell lines), which did not differ from their activities against the sensitive parent melanoma cell lines [Table 3]^[114]^.

Senescence in PDX BRAF-mutant MM27 melanoma cells was inhibited by deubiquitinase (DUB) USP7-mediated stabilization of RRM2. Suppression of USP7 led to cell growth inhibition and the formation of senescent cells (in line with an absence of apoptosis) via degradation of RRM2 and upregulation of HDAC activity. However, the senescent cells were eliminated by combined HDAC and LSD1 inhibition upon treatment with the dual HDAC/LSD1 inhibitor domatinostat (class I selective HDAC inhibitor). In BRAF-mutant MM27 PDX xenografts, the combination of domatinostat with the USP7 inhibitor P5091 was well-tolerated and inhibited melanoma growth accompanied by apoptosis induction and reduction of senescent cell numbers in the tumors [Table 3]^[115]^.

Immune evasive tumor modulation by chemotherapeutics is a major drawback in the therapy of various cancers. Class I HDAC inhibitors including panobinostat, entinostat, and mocetinostat enhanced immune therapy by durable induction of PD-L1 expression in various BRAF-mutant (WM983A, WM793) and BRAF-wildtype melanoma cells (B16F10) based on upregulated histone acetylation in the *PD-L1* gene which led to chromatin relaxation. *In vivo*, the combination of panobinostat with a PD-1 antibody led to reduced tumor growth and prolonged survival of B16F10-bearing mice [Table 3]^[116]^. BRAF^V600E^ PDX melanoma cells (Ma-Mel-55, Ma-Mel-86c, and Ma-Mel-86f cell lines) exposed to vemurafenib exhibited hampered NK cell recognition properties. Ligands such as MICA and CD155, which interact with NK activating receptors, were downregulated by vemurafenib. Treatment with sodium butyrate re-established NK cell recognition in the presence of vemurafenib through induction of MICA and nectin-2 [Table 3]. Thus, the combination of HDAC inhibition with BRAF inhibition can break the resistance of NK cells to melanoma cells^[117]^. Anaplastic thyroid cancer (ATC) is a rare but aggressive cancer with a bad prognosis. The HDAC inhibitors vorinostat and valproic acid induced G2/M cell cycle arrest in BRAF^V600E^ PF49 ATC cells and upregulated the expression of PD-L1 [Table 3]^[118]^. Thus, HDAC inhibition has the potential to sensitize BRAF-mutant ATC to checkpoint inhibitor therapy.

Since HDAC6 plays a vital role in immune evasive mechanisms, selective HDAC6 inhibitors turned out to be especially promising drug candidates for the treatment of various tumors [Table 3]^[15]^. The HDAC6 inhibitors nexturastat A and tubastatin A inhibited the proliferation of various BRAF-mutant and - wildtype melanoma cell lines (including the BRAF-mutant WM983A and WM793 cells) and augmented the antitumor immunity of treated tumor cells by upregulated expression of MHCI and melanoma antigens (gp100, MART1, TYRP1, and TYRP2)^[119]^. Nexturastat A was investigated more thoroughly in the BRAF^V600E^ SM1 melanoma model, where it leveled the increased PD-L1 expression upon anti-PD-1 therapy, promoted tumor infiltration by immune cells, and suppressed pro-tumorigenic M2-polarized macrophages. The combination of nexturastat A with an immune checkpoint inhibitor (anti-PD-L1) reduced SM1 tumor growth in mice more potently than the single drugs^[120]^. The nexturastat A-mediated inhibition of the conversion of anticancer M1-polarized macrophages to pro-tumorigenic M2-polarized was applied for further immune therapeutic strategies to treat BRAF-mutant melanomas, such as adoptive cell therapy with reprogrammed/nexturastat A-treated M1-polarized macrophages and anti-CD47 therapy^[121,122]^. Several new selective HDAC6 inhibitors (e.g., various nexturastat A analogs, the isoxazole SS208/AVS100, and the thiazole XP5) were disclosed, which displayed similar immunomodulating properties in melanomas^[123-127]^. Notably, the orally applicable HDAC6 inhibitor KS2507, which is structurally related to tubastatin A, underwent a clinical phase 1 study, which revealed stable disease in 7 out of 20 refractory solid tumor patients without dose-limiting toxicities^[128]^.

Independent from vemurafenib resistance, the curcumin-harmine-isovanillin combination drug GZ17-6.02 upregulated class I MHCA and suppressed PD-L1 in PDX BRAF^V600E^ melanoma cells, which was mediated by the downregulation of several HDACs (HDAC3, HDAC5, HDAC6, HDAC7, and HDAC8). The upregulation of MHCA and decreased expression of PD-L1 suggest an improved immune therapy response in BRAF-mutant melanoma treated with GZ17-6.02 [Table 3]^[105]^.

It is not surprising that pan-HDAC inhibitors can tackle a variety of cell death and survival mechanisms. The studies with more isoenzyme-selective inhibitors revealed that class I and IV HDACs, in particular, play an important role in the development of senescence by BRAF-mutant cancer cells. In addition, class I HDACs and HDAC6 (class IV) are crucial for the formation of tumor immunity.

### Sirtuins and cell death

Sirtuins have exerted manifold mechanisms to regulate the viability and cell death of BRAF-mutant cancers. A stunning example is the control of cellular acetyl-BRAF levels. The activation of BRAF by acetylation is regulated by p300 and SIRT1. Induction of p300 via RTK-Ras-AKT signaling leads to BRAF lysine K601 acetylation. Acetylated K601 of BRAF is a target of SIRT1, which downregulates the activity of BRAF by deacetylation [Table 4]. SIRT1 depletion in BRAF^V600E^ A375 cells led to vemurafenib resistance. Inhibition of p300 with C646 sensitized BRAF^V600E^ cells to vemurafenib indicated by reduced cell viability and fitness, while V600E/K601E-mutant HEK293 cells showed no sensitizing effects upon C646 treatment. Interestingly, the combination of the SIRT1 activator resveratrol with vemurafenib also reduced cell viability analogously to the described combination with the p300 inhibitor^[24]^.

**Table 4:** Sirtuins and their roles and functions in BRAF-mutant cancers

**Sirtuin**	**Mechanism(s)**	**Modulator(s)**
SIRT1	BRAF deacetylation, vemurafenib activity and resistance, stage- and cancer-dependent regulation of cell cycle, apoptosis, and metastasis, induction of autophagy (Beclin 1 deacetylation), proliferation upon SIRT1 upregulation, cancer-dependent senescence regulation	Resveratrol (activator), 4’-bromo-resveratrol (dual SIRT1/3 inhibitor), sirtinol (inhibitor)
SIRT2	BRAF/MEK inhibitor resistance upon SIRT2 suppression	-
SIRT3	Apoptosis induction (via caspase 3), cell cycle arrest (p21-mediated), metabolic reprogramming by dual SIRT1/3 inhibition	4’-bromo-resveratrol (dual SIRT1/3 inhibitor)
SIRT4	CRAF interaction and CRAF-MAPK suppression	-
SIRT5	Promotion of cell survival and proliferation, inhibition of apoptosis, expression of MITF and *c*-Myc	-
SIRT6	Apoptosis and autophagy (stage-dependent up- or downregulation), activation of ERK and Mcl-1, upregulation of LC3 and p62 and downregulation of AKT-mTOR, upregulation of IGF-1R and AKT in SIRT6-haploinsufficient cells	4*H*-chromene (activator), fluvastatin (activator), MDL-811 (activator), quercetin (inhibitor), quinazolinediones (inhibitors)
SIRT7	Promotion of cell survival, inhibition of apoptosis, upregulation of ERK, BRAF/MEK inhibitor resistance	-

MEK: MAPK/ERK kinase; ERK: extracellular signal-regulated kinase; MAPK: mitogen-activated protein kinase; BRAF: B-Raf, B-rapidly accelerated fibrosarcoma.

SIRT2 downregulation was also linked with BRAF/MEK inhibitor resistance in BRAF-mutant melanoma [Table 4]^[129]^. However, SIRT1 can act either as a tumor suppressor or as an oncogene, depending on the cell context. SIRT1 overexpression was initially associated with vemurafenib resistance in BRAF^V600E^ melanoma, and SIRT1 mediated MITF-induced melanoma proliferation. SIRT1 inhibition by sirtinol or EX-527 re-sensitized resistant BRAF-mutant melanoma cells to vemurafenib, and it was shown that SIRT1 downregulation promotes senescence, while upregulation promotes proliferation^[130]^. The dual SIRT1/SIRT3 inhibitor 4’-BR showed caspase 3-dependent apoptosis, p21-dependent cell cycle arrest, and metabolic reprogramming (decreased glucose uptake, lactate formation, and NAD^+^/NADH ratio) in melanoma cells [Table 4]^[131]^. 4’-BR inhibited melanoma growth and lung metastases in *Braf^V600E^*/*Pten^NULL^* mice without side effects by suppressing genes associated with tumor promotion (survivin and IGF1) and immune functions (IL1β and NLRP3)^[132]^. SIRT1 also deacetylates Beclin 1, leading to upregulated autophagy and degradation of E-cadherin accompanied by increased metastatic potential of BRAF-mutant melanoma cells^[133]^. In BRAF-mutant CRC, high levels of SIRT1 were associated with increased malignancy through suppression of apoptosis and senescence mechanisms^[134]^. Inhibition of SIRT1 was identified as a possible drug target in BRAF-altered CRC based on the *c*-Myc/NAMPT/SIRT1 feedback loop and *c*-Myc-mediated SIRT1 upregulation^[135]^. NAMPT inhibition induced apoptosis in BRAF^V600E^ CRC (HT-29 and COLO 205 cells), while SIRT1 inhibition with sirtinol required a combination with PI3K inhibition to induce cell death. Apoptosis induced by interference with the NAMPT-SIRT1 feedback loop was linked to *c*-Myc suppression and p53 activation^[136]^. Generally, SIRT1 acts in a tumor stage-dependent way. Tumor initiation at the pre-cancer stage is inhibited by SIRT1 based on increased DNA repair and genome stability, while SIRT1 promotes proliferation and survival at later stages of the tumor development, including metastasis and relapse by anti-apoptotic, pro-metabolism and anti-inflammatory mechanisms^[137]^.

BRAF/CRAF heterodimers are characteristic of class III BRAF mutations^[22]^. In addition, CRAF primarily activates ERK1/2 in NRAS-mutant melanoma^[138]^. The mitochondrial SIRT4 directly interacts with CRAF and suppresses CRAF-MAPK signaling accompanied by reduced p-ERK1/2 levels, which provides evidence for an extra-mitochondrial tumor-suppressing mechanism of SIRT4 [Table 4]^[139]^. Since HEK293 cells were used in this study, it remains to be determined how far this promising SIRT4 mechanism also applies to other BRAF-mutant cancer cell lines.

SIRT5 is responsible for the proliferation and survival of BRAF-mutant melanoma cells through repressing apoptosis as a consequence of chromatin modification and expression of survival factors such as MITF and *c*-Myc. Depletion of SIRT5 in vemurafenib-resistant cells inhibited cell proliferation, and caspase 3-dependent apoptosis was observed in several cutaneous and uveal melanoma cell lines upon SIRT5 suppression [Table 4]^[140]^. However, there is also evidence that SIRT5 is not required for BRAF^V600E^ melanoma growth *in vivo* and did not sensitize BRAF-mutant melanoma to BRAF inhibitor therapy^[141]^. This discrepancy can be explained by different *Sirt5* alleles used in these studies, and by possible differences in the applied mice populations^[140]^.

SIRT6 haploinsufficiency but not complete SIRT6 loss induced IGFBP2 expression followed by IGF-1R and AKT signaling activation in BRAF^V600E^ melanoma cells, leading to MAPK inhibitor resistance. The combination of the IGF-1R inhibitor linsitinib with the BRAF inhibitor dabrafenib suppressed the phosphorylation of IGF-1R and AKT and showed increased apoptosis induction in resistant SIRT6 haploinsufficient cells^[142]^. In BRAF^V600E^ K1 PTC cells, upregulated SIRT6 prevented apoptosis by activation of ERK and Mcl-1. Suppression of SIRT6 downregulated p-ERK and Mcl-1 while NF-κB and C-PARP were induced^[143]^. Autophagy is regulated by SIRT6 in BRAF-mutant melanoma dependent on the stage of the melanoma cells, and SIRT6 expression correlated with the expression of the autophagy factors LC3 and p62. SIRT6 was downregulated in primary melanoma cells but upregulated in cells from metastatic cell lines. While induced expression of SIRT6 in primary cells led to cell cycle arrest and apoptosis, upregulated SIRT6 in metastatic cells prevented apoptosis and promoted autophagy-mediated metastasis development. Mechanistically, SIRT6-catalyzed histone deacetylation suppressed IGF-1R, leading to inactivation of AKT-mTOR followed by induction of autophagy^[144]^. Similar to SIRT1, SIRT6 has been reported to exhibit both oncogenic and tumor-suppressing effects, and both activators (e.g., 4*H*-chromen, fluvastatin, MDL-800, and MDL-811) and inhibitors (e.g., quercetin and quinazolinediones) of SIRT6 can become useful anticancer drugs depending on the molecular biological context and stage of the treated tumor [Table 4]^[145]^.

Recently, the nuclear-localized SIRT7 deacetylase was correlated with acquired drug resistance in BRAF-mutant melanoma through the promotion of mitochondrial biogenesis. SIRT7 expression was upregulated by BRAF/MEK inhibitor therapy, while SIRT7 suppression sensitized BRAF-mutant cells to MAPK inhibitor therapy and led to increased apoptosis induction [Table 4]^[146]^. SIRT7 expression also upregulated ERK signaling in BRAF-mutant A375 melanoma cells under stress, which ensured cell survival^[147]^. The pro-oncogenic effects of SIRT7 are generally well described; however, there are also hints at tumor suppressor activities of SIRT7 at the cancer initiation stage^[148]^. How far such suppressing effects of SIRT7 also appear in BRAF-mutant tumors remains to be shown.

## CONCLUSION

HDACs (including sirtuins) have multiple effects on survival and drug resistance of BRAF-mutant cancers. While HDACs promote survival and inhibit various cell death mechanisms, inhibition of HDAC enzymes was reportedly pro-apoptotic and re-sensitized drug-resistant BRAF-mutant cancers to BRAF inhibitor and/or MEK inhibitor therapy. The underlying mechanisms comprise the regulation of the expression of apoptosis and survival-related genes (e.g., Bcl-2 family proteins) by histone acetylation/deacetylation, as well as the direct control of acetylation/deacetylation of non-histone substrates such as transcription factors, kinases (including BRAF K601 acetylation/deacetylation), and chaperones (e.g., Hsp90). Notably, senescent BRAF-mutant cells, which were resistant to cell death induced by BRAF inhibitor therapy, were also sensitized to MAPK inhibition therapy by HDAC inhibition. Autophagy is another important cellular catabolic mechanism found to be tightly regulated by HDACs, and autophagy-related factors (e.g., AMPK, beclin-1, AKT, mTOR) can be targeted to enhance BRAF and HDAC inhibition effects in BRAF-mutant cells.

The effects of HDACs on cell growth and survival were predominantly studied in BRAF^V600E^ melanoma models, but also other BRAF-altered cancers, such as colon cancer and thyroid cancer, revealed HDAC-dependent mechanisms of cell death. This underlines the great potential of HDAC inhibitors as promising combination partners for enhanced therapy of advanced and BRAF inhibitor-resistant cancers with activating BRAF mutations. Future studies focusing on the activity of HDAC inhibitors against non-melanoma cancers with mutant BRAF kinase have the potential to expand the scope of this promising class of anticancer drugs, as well as to enrich our knowledge of the influence of HDACs on Ras-MAPK signaling-mediated survival of cancer cells. The immunomodulatory effects of HDAC inhibitors suppressing immune evasion by BRAF-mutant tumors appear to be especially promising, given a growing number of immune therapeutics such as checkpoint inhibitors that have been approved for cancer therapy over the last few years. In particular, selective HDAC6 inhibitors exhibited remarkable immunomodulatory effects in BRAF-mutant cancers and enforced antitumor immunity and anticancer activity of checkpoint inhibitors.

## DECLARATIONS

### Copyright

© The Author(s) 2025.
